# Heat-induced blackening enhances antioxidants and reduces pungency in *Allium chinense*: Metabolomics insights

**DOI:** 10.1016/j.fochx.2026.104293

**Published:** 2026-08-12

**Authors:** Junxiong Hao, Yingying Yang, Jie Liu, Fei Shen, Ying Chen, Xiaoxia Zuo, Jing Wang, Liqin Zhu, Yonggen Shen, Rui Shi, Hongliang Yao, Libin Wang, Zhipeng Cai

**Affiliations:** a College of Food Science and Engineering, Jiangxi Agricultural University, Nanchang, Jiangxi 330045, China; b Department of Food Science and Technology, College of Light Industry and Food Engineering, Nanjing Forestry University, Nanjing, Jiangsu 210037, China; c College of Animal Science and Food Engineering, Jinling Institute of Technology, Nanjing, Jiangsu 210038, China; d National Key Laboratory of Crop Genetics and Germplasm Enhancement and Utilization, Sanya Institute of Nanjing Agricultural University, College of Horticulture, Nanjing Agricultural University, Nanjing, Jiangsu 210095, China; e Jiangxi Wanzai Food Processing Science and Technology Backyard, Yichun, Jiangxi 336100, China

**Keywords:** *Allium chinense*, Blackening, Heat treatment, Maillard reaction, Antioxidant capacity, Metabolomics, 5-HMF

## Abstract

Blackening of *Allium chinense* at 70–80 °C enhanced total acidity and reducing sugars, producing a pleasant sweet-sour flavor. Total phenolic content increased from 10.20 to 94.54 mg GAE/g at 75 °C, with enhanced antioxidant capacity (DPPH 66.73%, ABTS 90.59%, FRAP 113.40 μg AAE/g). Non-targeted metabolomics revealed marked reduction of γ-glutamyl-S-allyl-L-cysteine (GSAC) and related γ-glutamyl dipeptides, responsible for the characteristic pungency of fresh *A. chinense*, alongside upregulation of terpenoids, alkaloids, and flavonoids (2- to 5-fold). Blackening at 75 °C yielded optimal flavor, color, texture, and bioactivity, with 5-HMF accumulation (166 μg/g) within safe limits. This study provides insights into physicochemical and metabolic changes during blackening, offering a foundation for optimizing functional blackened *A. chinense* production.

## Highlights

• Heat-induced blackening transforms *Allium chinense* metabolic profiles.
• 75 °C maximizes phenolics and antioxidants while minimizing 5-HMF (166 μg/g).
• Metabolomics reveals heat significantly reduces pungent γ-glutamyl peptides.
• Beneficial terpenoids, alkaloids, and flavonoids are upregulated by heating.
• 75 °C optimizes blackened *Allium chinense* quality.

## 1 Introduction

*Allium chinense* (*A. chinense*) is an edible vegetable widely distributed in China, with considerable health and economic value attributed to its rich content of free amino acids and their derivatives, sugars, and minerals — these components collectively endow it with diverse flavor characteristics, as well as antioxidant and anti-inflammatory properties (S. Li et al., 2023). Beyond culinary uses, *A. chinense* also possesses medicinal properties: for instance, Li et al. reported that polysaccharides extracted from *A. chinense* possess positive antioxidant and hypoglycemic activities, while Qin et al. demonstrated that crude polysaccharides from *A. chinense* exert in vivo anti-atherosclerotic and hypolipidemic effects, along with a protective role against in vitro myocardial injury in rat cardiomyocyte (J. Li et al., 2025; Qin et al., 2024). In the food industry, *A. chinense* is predominantly processed via pickling to enhance its flavor and functional benefits (Lin et al., 2016). For example, Hou et al. utilized aged pickling brine to ferment *A. chinense*, yielding a fermented product with a short fermentation cycle and strong nitrite-reducing capacity (Hou et al., 2023). However, a major limitation of current processing methods is their failure to fully eliminate the pungent odor and taste of *A. chinense — a flaw that* restricts its public acceptance and thus limits consumption. Notably, garlic loses its pungency after heat-induced blackening while retaining its nutritional value.

Darkened products are a novel type of processed product obtained through high-temperature and high-humidity treatment. Their black color originates from the Maillard reaction between inherent reducing sugars and amino acids. The Maillard reaction is defined as a non-enzymatic condensation reaction between the carbonyl groups of reducing sugars and the amino groups of amino acids, peptides, or proteins. In recent years, the phenomenon of blackening has been extensively investigated in various food products, such as black garlic, black jujube, and black raspberry. This process significantly alters physicochemical, nutritional, and functional properties: blackened jujubes show increased total acid, polyphenols, reducing sugars, and 5-HMF, with stronger antioxidant activity (Y. Song et al., 2022); blackened blackberries contain higher anthocyanins and flavonols (H. Chen et al., 2017); and black garlic enhances cellular antioxidant defense and modulates energy metabolism pathways in cancer cells (Masztalerz et al., 2024).

The mechanistic effects of blackening have been extensively investigated in related products. In black garlic (*Allium sativum*), the Maillard reaction drives color, flavor, and bioactive compound formation, with temperature playing decisive roles in quality determination. Thermal processing induces marked changes in volatile sulfur compounds, free amino acids, polyphenols, and carbohydrates, with organosulfur compounds such as allicin undergoing thermal decomposition and enzymatic conversion. Notably, γ-glutamyl-S-allylcysteine (GSAC) levels decrease significantly during processing (Yoshimoto & Saito, 2019). In black jujube, similar temperature-dependent transformations have been documented, including increased polyphenols and 5-HMF alongside enhanced antioxidant activity (Y. Song et al., 2022). However, the mechanistic effects of blackening on *A. chinense* remain largely unexplored. This knowledge gap is significant because *A. chinense* possesses a distinct sulfur profile dominated by γ-glutamyl peptides rather than alliin, suggesting that its blackening-induced transformations may follow unique pathways.

Darkened products represent a promising category of functional foods, yet research on darkened *A. chinense* remains unexplored. Temperature during blackening modulates physicochemical properties, leading to quality variations. For example, garlic fermented at 90 °C yields bitter black garlic, whereas 70–75 °C produces high-nutritional-value black garlic with pleasant aroma, sweet flavor, and chewy texture (Chua et al., 2022). Given these temperature-dependent quality differences, it is necessary to investigate changes in physicochemical properties, antioxidant activity, and metabolomics of darkened *A. chinense* under different temperatures.

In the present study, changes in physicochemical properties and metabolite profiles of *A. chinense* during the blackening process were systematically investigated at 70 °C, 75 °C, and 80 °C. To our knowledge, this is the first report that integrally compares the effects of temperature on the quality attributes, antioxidant capacity, and non-targeted metabolomic landscape of blackened *A. chinense*. This study represents the first temperature-optimized blackening investigation of *A. chinense* using comprehensive non-targeted metabolomics; prior studies on Allium blackening have lacked systematic temperature optimization and sensory validation. Our work moves beyond descriptive analysis to provide a mechanistic understanding of how heat treatment simultaneously attenuates pungency and enhances antioxidant activity, addressing a major consumer acceptance barrier. Temperature selection was based on preliminary experiments and previous findings demonstrating that the optimal range for high-quality black garlic is 70–80 °C **(**Chua et al., 2022**)**, allowing systematic capture of the transition from suboptimal (70 °C) to optimal (75 °C) to potentially excessive (80 °C) conditions. Analyzed parameters included color, texture, total acid, reducing sugars, total phenols, antioxidant capacity, 5-HMF, and differential metabolites. This study aimed to identify the optimal temperature for *A. chinense* blackening and provide practical references for industrial production of high-quality blackened products.

## 2 Material and methods

### 2.1 Material and reagents

Fresh *A. chinense*s were purchased from a local supermarket in Nanchang, Jiangxi Province, China (GPS coordinates: 28.6820°N, 115.8579°E). The plant material was authenticated by Dr. Liqin Zhu (College of Food Science and Engineering, Jiangxi Agricultural University) based on morphological characteristics following the Flora of China taxonomic descriptions. A voucher specimen (JXAU-AC-2024-001) has been deposited in the Herbarium of the College of Food Science and Engineering, Jiangxi Agricultural University. 5-HMF (≥ 99% purity), acetonitrile (HPLC), formic acid (HPLC) were purchased from Shanghai Aladdin Biochemical Technology Co., Ltd. 2,2-Diphenyl-1-picrylhydrazyl (DPPH (≥ 98% purity)), 2′-azido-bis(3-ethylbenzothiazoline-6-sulfonic acid) (ABTS (≥ 98% purity)), 2,4,6-tris(2-pyridinyl)-*s*-triazine (TPTZ (≥ 98% purity)), and salicylic acid were purchased from Shanghai Macklin Biochemical Technology Co., Ltd. (Shanghai, China). All other chemical reagents were of analytical grade or higher.

### 2.2 Preparation of black *Allium chinense*

Fresh *A. chinense* bulbs were cleaned and placed in high-temperature-resistant No. 10 sealing bags (approximately 47–50 bulbs per bag, 520 g ± 5 g). The sealed bags were then subjected to blackening in a constant temperature oven at 70 °C, 75 °C, and 80 °C for 10 days. During processing, samples remained inside sealed bags without additional humidity control. For each temperature condition, three independent production batches were prepared (*n* = 3), corresponding to three sealed bags per temperature group. Blackened samples were quick-frozen and ground in liquid nitrogen, and placed at −80 °C for subsequent analysis.

### 2.3 Determination of the degree of browning

Browning degree was determined using a colorimeter (ColorQuest XE, Hunter Lab Instruments Co., Ltd., USA), following Zheng et al. (2024) with minor modifications. Samples were collected every 2 days from each temperature group, and their color parameters (L*, a*, b*) were measured using the colorimeter. Each measurement was performed in triplicate to ensure data reliability. The total color difference (ΔE) was calculated using the following formula:

(1) $$∆E=\sqrt{{\left(L_{1}-L_{0}\right)}^{2}+{\left(a_{1}-a_{0}\right)}^{2}+{\left(b_{1}-b_{0}\right)}^{2}}$$

where L_0_, a_0_, b_0_ values denote fresh *A. chinense*, L_1_, a_1_, b_1_ denote blackened samples.

### 2.4 Determination of water content

The moisture content (MC) of *A. chinense* samples was determined following the direct drying method (Šumić et al., 2023). The specific procedure was as follows: Accurately weigh 2 g of *A. chinense* samples subjected to different blackening treatments, and spread the samples evenly in a pre-dried and pre-weighed aluminum weighing dish. Subsequently, place the weighing dish in a forced-air drying oven and dry the samples at a constant temperature of 105 °C. Continue drying until the samples reach a constant weight.

### 2.5 Determination of hardness

Texture profile analysis (TPA) was performed on *A. chinense* samples in accordance with the protocol outlined by Masztalerz et al., using a TA.XT Plus texture analyzer (Stable Micro Systems, Surrey, UK) (Masztalerz et al., 2024). The parameters for the TPA were configured as follows: a P/6 probe was chosen, with the pre-test, test, and post-test speeds all set to 1 mm/s.

### 2.6 Determination of pH and total acid

The total acid (TA) content of *A. chinense* samples was determined via potentiometric titration following Ma et al. with minor modifications (Ma et al., 2021). Briefly, 5 g of *A. chinense* samples were accurately weighed. Each weighed sample was mixed with distilled water and made up to a final volume of 50 mL in a volumetric flask; the mixture was then homogenized thoroughly to ensure full extraction of acids. The homogenized solution was centrifuged at 8000*g* for 15 min, and the supernatant was filtered through qualitative filter paper to remove solid residues. A 20 mL aliquot of the filtrate was transferred to a new volumetric flask and diluted to 50 mL with distilled water to adjust the acid concentration within the optimal detection range. For titration, the diluted filtrate was transferred to a beaker, and a pH electrode connected to a potentiometer was inserted into the solution. The solution was magnetically stirred at 300 g, and titrated with 0.05 mol/L sodium hydroxide (NaOH) standard solution until the pH reached 8.2. The initial pH value of the diluted filtrate and the total volume of NaOH consumed at the endpoint were recorded. The TA content of the sample was calculated using the following formula:

(2) $$\mathrm{TA}\;\left(\%\right)=\left(C\times \left(V-V_{0}\right)\times 0.09\times V_{2}\times 100\right)/\left(V_{1}\times m\right)$$

where C is the molarity of NaOH standard solution (mol·L^−1^); V and V_0_ represent the consumed volumes of NaOH for sample titration and blank test (mL), respectively; 0.090 is the conversion coefficient for lactic acid (g/mmol); V₁ is the volume of the aliquot taken for secondary dilution (20 mL); V₂ is the final volume after secondary dilution (50 mL); and m is the sample weight (g).

### 2.7 Determination of 5-HMF content

5-HMF content in blackened *A. chinense* samples was determined via liquid chromatography (LC, Nexera LC-40, Shimadzu Corporation, Kyoto, Japan), with the method slightly modified from J. Liu et al. (2024). A 5-HMF standard stock solution (1.0 mg/mL) was prepared by dissolving 10.0 mg of standard (≥ 99% purity) in 10 mL of methanol. Working standard solutions at 0.5, 2.5, 5.0, 25.0, 50.0, and 100.0 μg/mL were prepared by serial dilution with methanol and stored at 4 °C. Accurately weigh 1 g of blackened *A. chinense*, grind thoroughly, transfer to a 50 mL volumetric flask, add 3 mL of 10.6% potassium ferrocyanide solution and 3 mL of 21.9% zinc acetate solution (for protein precipitation), shake vigorously, bring to volume with methanol, stand at room temperature for 30 min, centrifuge at 8000 *g* for 15 min, and filtered through qualitative paper and a 0.22 μm syringe filter. LC conditions: Inertsustain® C18 column (4.6 mm × 250 mm, 5 μm), column temperature 35 °C, 20 min runtime; mobile phase was 0.1% formic acid in water: acetonitrile (95: 5, v / v), flow rate was 1.0 mL/min (isocratic elution), injection volume was 10 μL, detection wavelength was 284 nm. The calibration curve showed R^2^ > 0.999. Representative HPLC chromatograms are provided in Supplementary Fig. S1.

### 2.8 Determination of reducing sugars

The reducing sugar content in *A. chinense* samples (from different temperature groups and blackening time points) was determined following the methodology of Qiu et al. (Qiu et al., 2024): Accurately weigh 5 g of each sample, add distilled water to a final volume of 50 mL, stand for 30 min, centrifuge at 8000 *g* for 15 min, filter the supernatant through qualitative filter paper (reserve the filtrate for analysis); then mix 0.2 mL of the filtrate with 0.8 mL of 3,5-dinitrosalicylic acid (DNS) solution and 0.2 mL of distilled water, heat in a boiling water bath for 10 min, cool rapidly, dilute with distilled water to 2 mL, measure the absorbance at 540 nm using a spectrophotometer (Tsinghua Tongfang Co., Ltd.), and calculate the reducing sugar content based on a standard curve prepared with gradient glucose solutions.

### 2.9 Determination of total phenols

Total phenolic content was determined using the Folin-Ciocalteu method (Y.-A. Chen et al., 2018). Accurately weigh 5 g of samples (from different temperature groups and blackening time points), centrifuge at 8000 *g* for 15 min, filter the supernatant through qualitative filter paper; then mix 0.2 mL of the filtrate with 0.1 mL of Folin-Ciocalteu reagent and 0.3 mL of 10% sodium carbonate solution, add water to a final volume of 2 mL, incubate in a 75 °C water bath for 10 min, let stand at room temperature for 2 h, then determine the absorbance at 760 nm with a spectrophotometer, and compute the total phenolic content by reference to a standard curve created using gallic acid solutions of gradient concentrations.

### 2.10 Determination of antioxidant capacity

#### 2.10.1 DPPH analysis

The DPPH radical scavenging rate of samples was determined based on a previous methodology (Chang et al., 2023). Take 1 mL of 100 mg/mL centrifugation solution (prepared from samples at different temperatures and time points), dilute to 50 mL with anhydrous ethanol; mix 100 μL of this diluted solution with 100 μL of 0.02 mmol/L DPPH solution and add to a 96-well microplate, with a control group prepared by mixing 100 μL of anhydrous ethanol and 100 μL of 0.02 mmol/L DPPH solution; incubate the microplate at room temperature for 30 min in the dark, then measure the absorbance at 517 nm, and calculate the DPPH radical scavenging rate using the following equation:

(3) $$\text{DPPH radical scavenging capacity}\;\left(\%\right)=\left[\left(A_{0}-A_{1}\right)/A_{0}\times 100\%\right]$$

where A_0_ is the absorbance of the control (ethanol) and A_1_ is the absorbance of the sample.

#### 2.10.2 ABTS test

The ABTS radical scavenging rate was determined with reference to a previous methodology (H. Yu et al., 2025). Mix 7 mmol/L ABTS solution with 4.9 mmol/L potassium persulfate, and incubate in the dark for 24 h to prepare ABTS stock solution; dilute the ABTS stock solution with 0.1 mol/L phosphate buffer (pH 7.4) to adjust its absorbance at 734 nm to 0.7, yielding ABTS working solution; mix 50 μL of 100 mg/L sample solution with 200 μL of ABTS working solution in a 96-well microplate, incubate in darkness for 10 min, and subsequently determine the absorbance at 734 nm with a microplate reader (MultiScan MK3, Thermo Fisher Scientific, USA); use an equal volume of distilled water instead of sample solution as the blank control, and calculate the ABTS radical scavenging rate as per the equation below:

(4) $$\text{ABTS radical scavenging ability}\%=\left(1-A_{1}/A_{0}\right)\times 100\%$$

where A_1_ is the absorbance of the sample and A_0_ is the absorbance of the control (distilled water).

#### 2.10.3 Analysis of hydroxyl radical scavenging rate

The hydroxyl radical scavenging capacity was determined with slight modifications to a previous methodology (F. Song et al., 2024). Mix 0.05 mL of 100 mg/mL sample solution with 0.05 mL of 9 mmol/L FeSO₄, 0.05 mL of 9 mmol/L salicylic acid solution, and 1 mL of 9 mmol/L H₂O₂ solution, then measure the absorbance (A₁) at 510 nm; set two control groups: one with an equal volume of distilled water replacing the salicylic acid solution (absorbance A₂) and another with an equal volume of distilled water replacing the sample solution (absorbance A₀), and calculate the hydroxyl radical scavenging capacity according to the following formula:

(5) $$\text{Hydroxyl radical scavenging rate}/\%=\left[1-\left(A_{1}-A_{2}\right)/A_{0}\right]$$

#### 2.10.4 FRAP assay

The ferric reducing antioxidant power (FRAP) was determined based on a previous method (F. Song et al., 2024). Prepare the FRAP solution by mixing 300 mmol/L acetate buffer, 10 mmol/L 2,4,6-tripyridyl-*s*-triazine (TPTZ) solution, and 20 mmol/L FeCl₃ solution at a volume ratio of 10: 1: 1 (v / v / v); mix 10 μL of sample solution with 300 μL of FRAP solution, stand at room temperature for 30 min, then measure the absorbance at 593 nm, and quantify the FRAP value using a standard curve prepared with gradient ascorbic acid solutions.

### 2.11 Non-target metabolomics analysis

For metabolomic analysis, 25 mg of the previously prepared solid *A. chinense* sample was weighed into an EP tube under low-temperature conditions; homogenization beads and 1000 μL of extraction solution (methanol: acetonitrile: water = 2: 2: 1, v / v / v, containing isotope-labeled internal standards) were added, followed by vortex mixing for 30 s. The tube was placed in a homogenizer for homogenization (35 Hz, 4 min), then transferred to an ice-water bath for ultrasonic treatment (5 min), with this homogenization-ultrasonication cycle repeated 3 times. After static incubation at −40 °C for 1 h, 400 μL of the mixture was added to the wells of a 0.22 μm filter plate; the protein precipitation plate-collection plate assembly was placed in a positive pressure device (6 psi) for filtration, and this filtration step was repeated 3 times. The sample was then statically incubated at −40 °C for another 1 h, after which 400 μL of the mixture was again added to a 0.22 μm filter plate and filtered in the positive pressure device (6 psi, 120 s); the filtrate was collected for instrumental analysis. Quality control (QC) samples were prepared by mixing equal volumes of supernatant from all samples, and these were also analyzed alongside the test samples. Chromatographic separation of target compounds was performed on a Phenomenex Kinetex C18 column (2.1 mm × 50 mm, 2.6 μm) using a Vanquish ultra-performance liquid chromatograph (UPLC, Thermo Fisher Scientific), with mobile phase A as water containing 0.01% acetic acid and mobile phase B as isopropanol: acetonitrile (1: 1, v / v); the sample plate temperature was maintained at 4 °C, and the injection volume was 2 μL. Detection was carried out using an Orbitrap Exploris 120 mass spectrometer (Thermo Fisher Scientific) with the following parameters: sheath gas flow rate = 50 Arb, aux gas flow rate = 15 Arb, capillary temperature = 320 °C, full MS resolution = 60,000, MS / MS resolution = 15,000, collision energy = SNCE 20 / 30 / 40, spray voltage = 3.8 kV (positive ion mode) or − 3.4 kV (negative ion mode) (Z. Zhou et al., 2022).

Raw data were processed using Compound Discoverer 3.3 software with TIC-based normalization. Features with >50% missing values across all samples or RSD ≥ 30% in QC samples were removed. Missing values were imputed using k-NN (k = 5). Metabolite annotation was performed against HMDB, METLIN, and an in-house library, with confidence levels assigned according to MSI criteria (Levels 1–4).

### 2.12 Statistical analysis

All experimental data were obtained from three independent replicates. Data analysis was performed using SPSS Statistics 27.0 software. Prior to one-way analysis of variance (ANOVA), the assumptions of normality (Shapiro–Wilk test) and homogeneity of variance (Levene's test) were verified; data were log-transformed when necessary to meet these assumptions. One-way ANOVA was applied for statistical analysis, followed by Tukey's honestly significant difference (HSD) post-hoc test for multiple comparisons, and results were expressed as mean ± standard deviation (SD) of three replicates. Graphs were generated using GraphPad Prism 8, Origin 2024, and Omic Studio tools. Principal component analysis (PCA) and orthogonal partial least squares discriminant analysis (OPLS-DA) were conducted using the ropls R package (v1.22.0). The OPLS-DA models were validated using 200 permutation tests to assess the risk of overfitting. The model parameters were as follows: 70 °C group: R^2^X = 0.762, R^2^Y = 0.999, Q^2^ = 0.996; 75 °C group: R^2^X = 0.820, R^2^Y = 0.999, Q^2^ = 0.997; 80 °C group: R^2^X = 0.824, R^2^Y = 0.999, Q^2^ = 0.998. Hierarchical clustering analysis heatmaps were constructed with the pheatmap R package (v1.0.12); Kyoto Encyclopedia of Genes and Genomes (KEGG) enrichment analysis was performed via the clusterProfiler R package (v3.18.1). For differential metabolite analysis, metabolites were screened using the following criteria: Student's *t*-test *p* ≤ 0.05, Variable Importance in the Projection (VIP) ≥ 1.0 from the OPLS-DA model, and fold change (FC) ≥ 2.0 or ≤ 0.5. To control the false discovery rate, the Benjamini–Hochberg correction was applied, and metabolites with adjusted p (q) ≤ 0.05 were considered significantly different.

## 3 Results and discussion

### 3.1 Browning of *A. chinense* during the blackening process

**Fig. 1:**
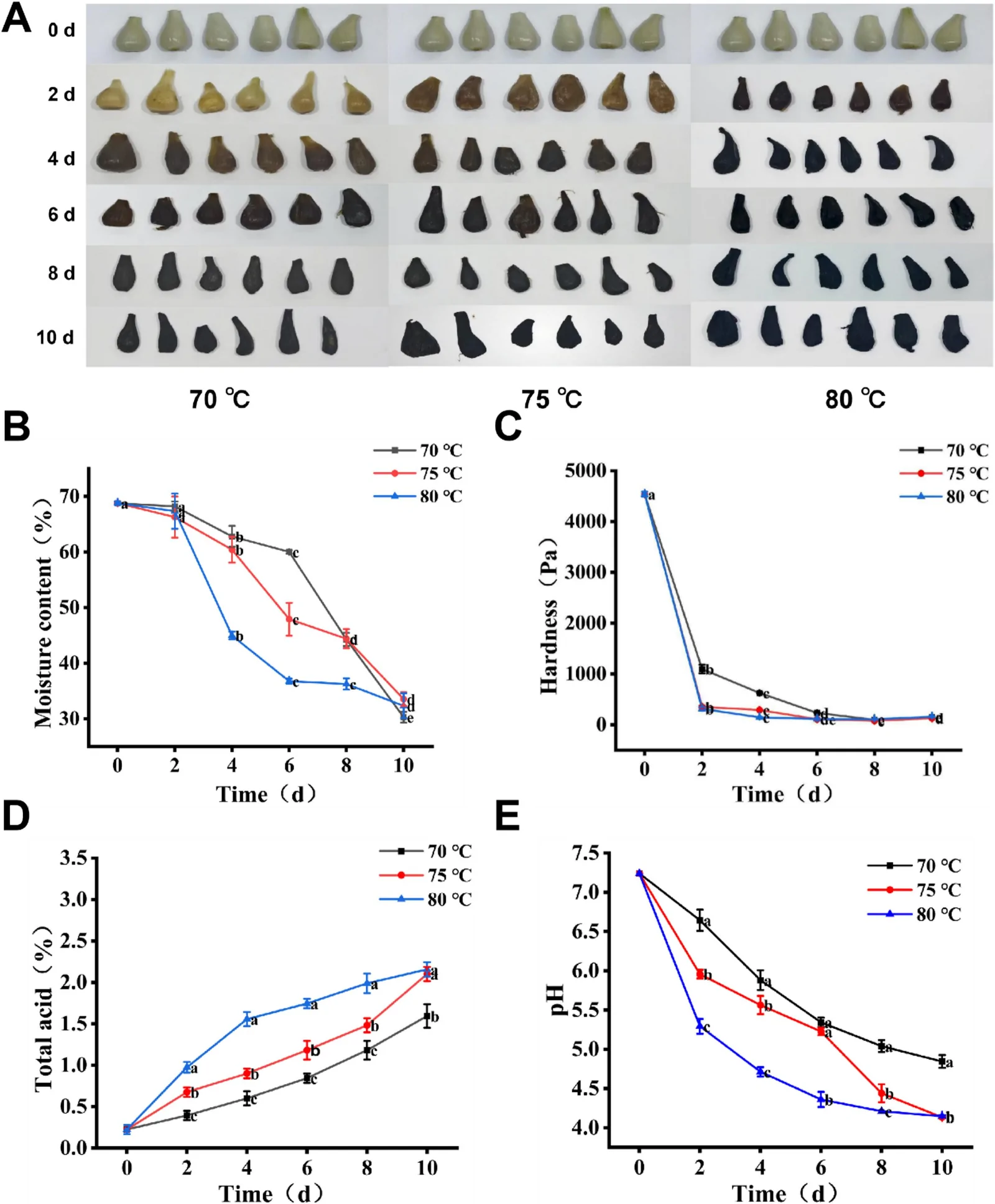
Changes in physicochemical properties of *A. chinense* during blackening at different temperatures (70 °C, 75 °C, and 80 °C). (A) Visual color changes of samples during blackening; (B) Changes in water content (%, mean ± SD, *n* = 3); (C) Changes in hardness (Pa, mean ± SD, n = 3); (D) Changes in total acid content (, % lactic acid, mean ± SD, n = 3); (E) Changes in pH value (mean ± SD, n = 3). Error bars represent standard deviation (SD) from three independent replicates (n = 3).

The color evolution of *A. chinense* during heat treatment, a critical visual quality indicator, is summarized in Table 1 and Fig. 1A. With prolonged treatment time, the L* value (lightness) decreased, reaching minimum values of 31.36 on day 10 at 70 °C, 29.58 on day 10 at 75 °C, and 30.53 on day 4 at 80 °C; notably, the L* values after treatment at all three temperatures were significantly lower than those of fresh *A. chinense* (*p* < 0.05), which may be associated with melanin formation via the Maillard reaction at elevated temperatures (Tsai et al., 2021). Differences in a* and b* values were observed between temperature groups, potentially attributed to temperature-specific biochemical changes. ΔE values reached maxima of 46.57 (70 °C, day 10), 48.73 (75 °C, day 10), and 47.77 (80 °C, day 4, then slightly decreased). These trends align with Yang et al. (2023) and Liu et al. (2021), who reported that higher temperatures promote browning reactions and faster color changes—phenomena driven by non-enzymatic browning. (Y. Liu et al., 2021; N. Yang et al., 2023). Notably, the ΔE values were associated with 5-HMF accumulation, suggesting that color development during blackening was closely related to the Maillard reaction.

**Table 1:** Color changes during blackening.

Temperature	Time	L*	a*	b*	∆E
70 °C	Day0	77.12 ± 0.31^a^	−1.01 ± 0.05^d^	9.88 ± 0.08^c^	0^f^
	Day2	61.43 ± 0.12^b^	−0.68 ± 0.59^d^	14.45 ± 1.44^b^	16.39 ± 0.43^e^
	Day4	46.32 ± 0.56^c^	3.79 ± 0.75^bc^	5.03 ± 0.51^d^	33.31 ± 0.15^d^
	Day6	42.56 ± 0.69^d^	7.95 ± 0.84^a^	18.82 ± 0.74^a^	35.24 ± 0.72^c^
	Day8	32.93 ± 0.29^e^	4.86 ± 0.36^b^	5.16 ± 0.09^d^	44.83 ± 0.31^b^
	Day10	31.36 ± 0.40^f^	3.56 ± 0.27^c^	2.54 ± 0.33^e^	46.57 ± 0.34^a^

75°C	Day0	77.12 ± 0.31^a^	−1.01 ± 0.05^d^	9.88 ± 0.08^a^	0^e^
	Day2	45.19 ± 0.22^b^	3.8 ± 0.99^a^	8.76 ± 1.68^a^	32.35 ± 0.18^d^
	Day4	34.45 ± 0.45^c^	5.31 ± 2.31^a^	5.73 ± 2.36^b^	43.42 ± 0.38^c^
	Day6	30.95 ± 0.26^d^	4.63 ± 0.61^b^	2.82 ± 0.51^c^	47.05 ± 0.25^b^
	Day8	30.62 ± 0.32^d^	1.55 ± 0.32^b^	1.01 ± 0.14^cd^	47.41 ± 0.33^b^
	Day10	29.58 ± 0.32^e^	0.17 ± 0.07^c^	−0.78 ± 0.21^d^	**48.73** **±** **0.25**^**a**^

80 °C	Day0	77.12 ± 0.31^a^	−1.01 ± 0.05^c^	9.88 ± 0.08^a^	0^d^
	Day2	36.24 ± 1.31^c^	4.99 ± 2.01^a^	5.34 ± 2.99^b^	41.66 ± 1.48^b^
	Day4	30.53 ± 0.28^d^	0.57 ± 0.37^b^	−0.57 ± 0.32^c^	47.77 ± 0.32^a^
	Day6	40.14 ± 0.24^b^	0.32 ± 0.14^bc^	−0.71 ± 0.16^c^	38.49 ± 0.20^c^
	Day8	40.73 ± 0.07^b^	1.29 ± 0.23^b^	1.05 ± 0.18^c^	37.51 ± 0.12^c^
	Day10	41.17 ± 0.86^b^	0.62 ± 0.02^b^	−0.44 ± 0.50^c^	37.42 ± 0.98^c^

**Note:** Data are expressed as mean ± SD (n = 3). Day 0 represents fresh (non-blackened) *A. chinense*. Different superscript letters in the same column within each temperature group indicate significant differences (p < 0.05).

### 3.2 Changes in moisture content

Moisture content is critical for blackened *A. chinense* quality: too low causes hard texture; too high causes softening and spoilage. As shown in Fig. 1B, moisture content decreased significantly at all three temperatures compared to fresh samples (p < 0.05), with faster decreases at higher temperatures. Moisture declined from an initial 68.77% to 30.30% (70 °C, day 10), 33.55% (75 °C, day 10), and 32.34% (80 °C, day 10). This trend aligns with Diamante et al. (2012) and J. Liu et al. (2025), who attributed moisture loss to temperature-dependent water migration. Nungwongsa et al. (2021) proposed that higher temperatures increase the temperature gradient, elevating heat transfer and moisture diffusion coefficients, accelerating moisture loss. Lower moisture content inhibits microbial growth, extending shelf life, and promotes browning reactions (T. Chen et al., 2024).

### 3.3 Variation in hardness

During the heat treatment of *A. chinense*, a substantial change in its texture was observed, resulting in a texture that is analogous to that of preserved fruit. As shown in Fig. 1C, the hardness of *A. chinense* decreased significantly with prolonged treatment time at all three temperatures (70, 75, and 80 °C), starting from an initial value of 4538.05 g. Specifically, at 70 °C treatment, the hardness decreased to 1096.68 g on day 2 and reached a minimum of 96.68 g on day 8; at 75 °C treatment, it declined to 352.03 g on day 2 and reached the lowest value of 79.15 g on day 8; at 80 °C treatment, it dropped to 313.39 g on day 2 and reached a minimum of 106.10 g on day 8. Notably, hardness decreased more rapidly at higher temperatures, which is consistent with the hardness change pattern observed during the aging process of black garlic, as reported by Shin et al. (2024).

### 3.4 Changes in pH and total acid

Fig. 1D and E illustrates the effects of different heat treatment temperatures on the total acid content and pH value of *A. chinense*. At each temperature (70, 75, and 80 °C), the pH value of the sample solution decreased significantly over time (*p* < 0.05), while the total acid content increased significantly (p < 0.05). Specifically, at 70 °C, the total acid content increased by 6.08-fold, and the pH value decreased from an initial 7.24 to 4.84; at 75 °C, the total acid content increased by 8.3-fold, with the pH value dropping from 7.24 to 4.13; at 80 °C, the total acid content rose by 8.58-fold, and the pH value decreased from 7.24 to 4.15. This trend can be attributed to the abundant organic acids generated via the Maillard reaction during the blackening process of *A. chinense*. Importantly, the decrease in pH not only enhanced the storage stability of *A. chinense* products but also contributed to the development of a desirable acidic flavor, which aligns with previous findings reported by Z. Zhang et al. (2015).

### 3.5 Changes in the content of 5-HMF

**Fig. 2:**
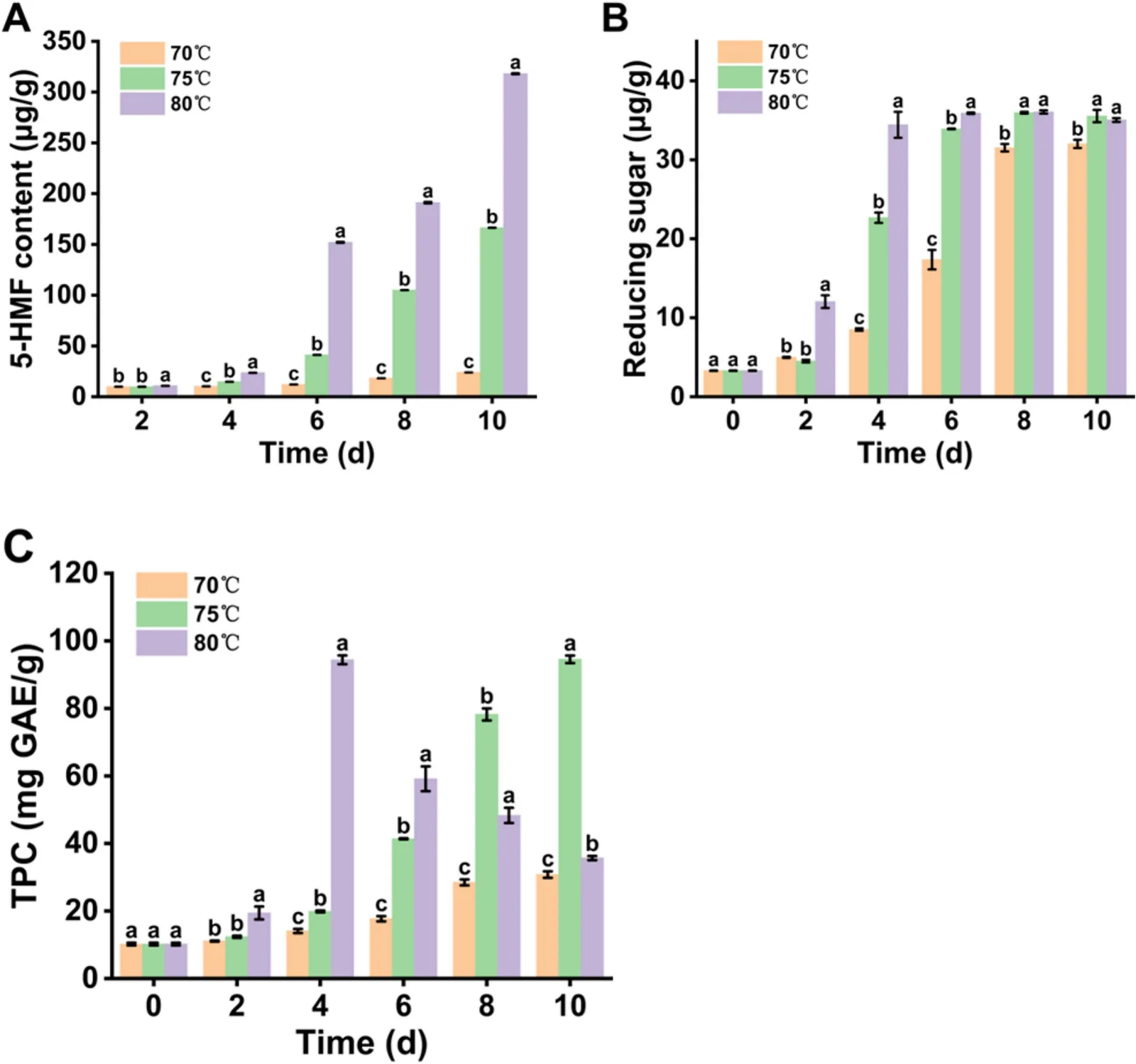
Changes in chemical components of *A. chinense* during blackening at different temperatures (70 °C, 75 °C, and 80 °C). (A) Changes in 5-hydroxymethylfurfural (5-HMF) content (μg/g, mean ± SD, n = 3); (B) Changes in reducing sugar content (μg/g, mean ± SD, n = 3); (C) Changes in total phenolic content (mg GAE/g, mean ± SD, n = 3). Error bars represent standard deviation (SD) from three independent replicates (n = 3).

**Fig. 3:**
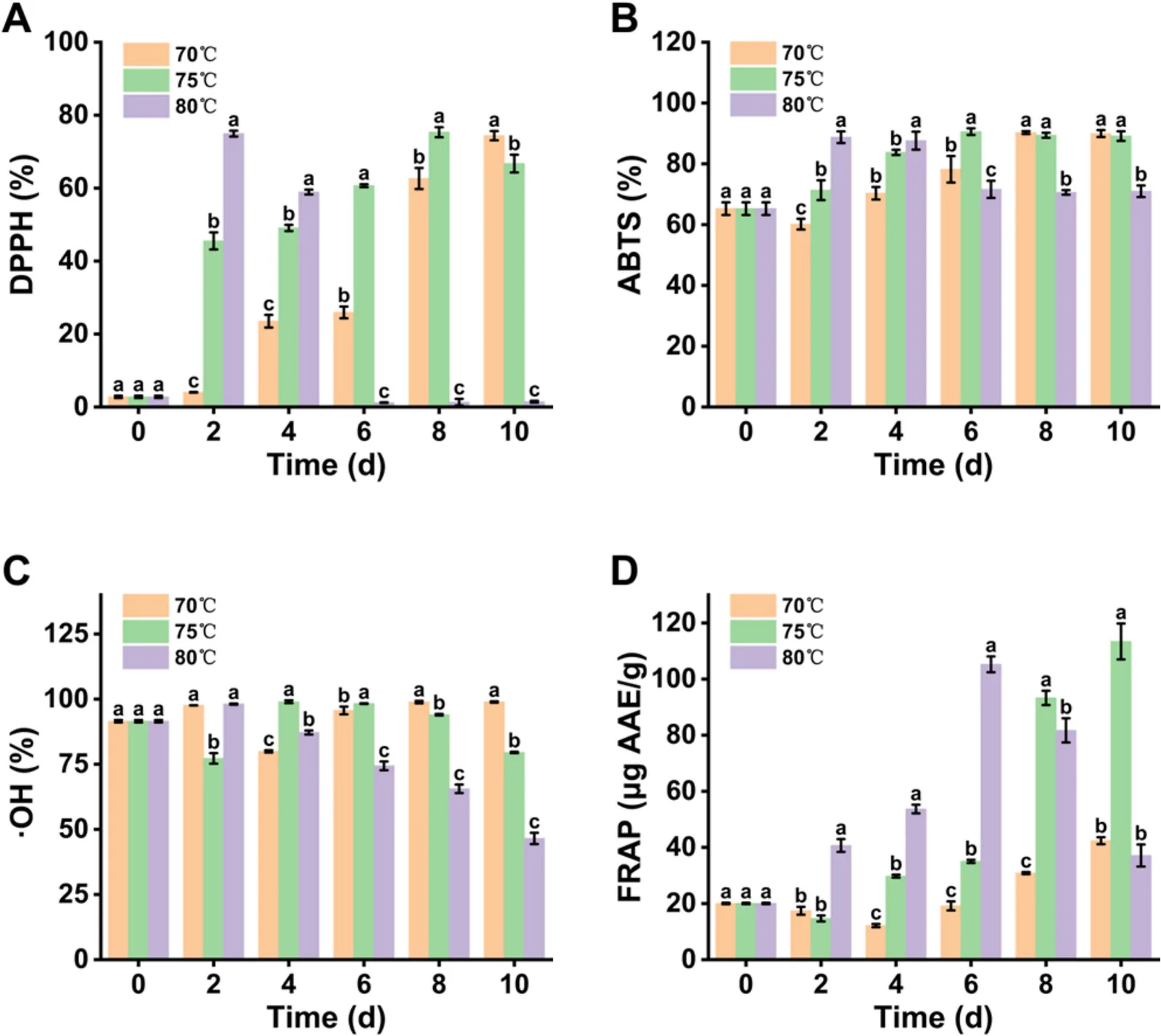
Antioxidant capacity of *A. chinense* during blackening at different temperatures (70 °C, 75 °C, and 80 °C). (A) DPPH free radical scavenging rate (%, mean ± SD, n = 3); (B) ABTS free radical scavenging rate (%, mean ± SD, n = 3); (C) Hydroxyl radical scavenging rate (%, mean ± SD, n = 3); (D) FRAP ferric reducing antioxidant power (μg AAE/g, mean ± SD, n = 3). Error bars represent standard deviation (SD) from three independent replicates (n = 3).

5-HMF is a major intermediate of the Maillard reaction (R. Zhou et al., 2024). The identification and quantification of 5-HMF were performed by HPLC with a C18 column and UV detection at 284 nm. Representative chromatograms of 5-HMF standards and blackened *A. chinense* samples are shown in Supplementary Fig. S1. As shown in Fig. 2A, 5-HMF content increased significantly with treatment time (p < 0.05), with accelerating rates—consistent with Y. Chen et al. (2019). Specifically, 5-HMF reached maxima of 23.89 μg/g (70 °C), 166.37 μg/g (75 °C), and 318.14 μg/g (80 °C), indicating higher temperatures promoted faster 5-HMF accumulation. 5-HMF content is influenced by temperature, pH, and sugar content (Nguyen et al., 2017). For instance, 5-HMF is generated under acidic conditions, and its content increases with rising acidity — this aligns with the results presented in Fig. 2C and D and Fig. 3B of this study, further supporting the observed accumulation of 5-HMF in heat-treated *A. chinense*. The formation of 5-HMF is primarily achieved through the Maillard reaction, making it a valid indicator for the heat-induced blackening reaction of *A. chinense* (Manickavasagam et al., 2023).

To assess the safety of the 5-HMF levels in our products, we compared our results with established limits. The 5-HMF content at 80 °C (318.14 μg/g = 0.318 mg/g) was substantially below the black garlic safety limit (≤ 3.04 mg/g) (Chang et al., 2023). However, it exceeded the EU honey guideline of 15 mg/kg (15 μg/g; EU Council Directive 2001/110/EC), though this standard is specific to honey and not directly applicable to processed vegetables. Importantly, based on the JECFA tolerable daily intake of 0–0.54 mg/kg body weight for 5-HMF, the estimated daily intake from consuming 100 g of 75 °C-blackened *A. chinense* (approx. 16.6 mg) would be well within the safe limit. From a toxicological perspective, the no observed adverse effect level (NOAEL) of 5-HMF in animal studies ranges from 80 to 100 mg/kg body weight, far exceeding the estimated intake from our product. While in vitro genotoxicity has been observed under specific conditions, in vivo studies have been negative, and long-term carcinogenicity studies showed no tumor induction. To further minimize 5-HMF accumulation, future research could explore mitigation strategies such as stepwise heating protocols or optimization of processing parameters.

### 3.6 Changes in reducing sugars

The reducing sugar content of *A. chinense* treated at different temperatures is presented in Fig. 2B. As heat treatment duration prolonged, the reducing sugar content exhibited a significant increase (*p* < 0.05) across all tested temperatures, with a faster increase rate observed at higher temperatures. Specifically, at 70 °C, the reducing sugar content rose from an initial 3.29 μg/g to a maximum of 32.01 μg/g; at 75 °C and 80 °C, it increased to peak values of 35.96 μg/g and 36.05 μg/g, respectively, both on day 8. This initial increase is likely attributed to the breakdown of polysaccharide carbon chains into reducing sugars under high-temperature conditions. Notably, after day 8, the reducing sugar content decreased slightly at 75 °C and 80 °C, which may be explained by the consumption rate of reducing sugars in the Maillard reaction exceeding their accumulation rate at these temperatures. Reducing sugars exhibit high solubility and sweetness, and the increase in their content endows blackened *A. chinense* with a certain degree of sweetness (Tena et al., 2025). The key to the blackening of *A. chinense* lay in the Maillard reaction, in which reducing sugars serve as the main reactants. An increase in reducing sugars intensifies the Maillard reaction, thereby enhancing the color development and generating unique flavors of blackened *A. chinense*.

### 3.7 Changes in total phenolic content

The effect of different temperature treatments on the total phenol content (TPC) of *A. chinense* is shown in Fig. 2C. At all tested temperatures, the TPC of heat-treated samples was notably higher than that of the untreated sample (*p* < 0.05), a finding that aligns with earlier studies (Lu et al., 2017). Specifically, at 70 °C treatment, the TPC reached a maximum of 30.78 mg gallic acid equivalents per gram (mg GAE/g) on day 10; at 75 °C treatment, it peaked at 94.54 mg GAE/g on day 10; and at 80 °C treatment, it reached the highest value of 94.38 mg GAE/g on day 4. Notably, after reaching the maximum at 80 °C, the TPC began to decrease—this phenomenon may be attributed to the thermal degradation of phenolic compounds and excessive Maillard reaction progression. At elevated temperatures, the Maillard reaction generates not only intermediate products with antioxidant activity but also advanced glycation end-products (AGEs) and melanoidins that may not possess the same radical-scavenging capacity as intermediate Maillard reaction products. Furthermore, prolonged heating at 80 °C can induce the polymerization and condensation of phenolic compounds into high-molecular-weight complexes with reduced extractability and bioactivity (Lang et al., 2019). These combined effects—thermal degradation, over-polymerization, and the formation of less bioactive advanced glycation products—collectively account for the decline in TPC observed at 80 °C.

### 3.8 Changes in antioxidant capacity

The effect of different temperature treatments on the DPPH radical scavenging rate of *A. chinense* is shown in Fig. 3A. At 70 and 75 °C, the DPPH radical scavenging rate was significantly higher than that of fresh *A. chinense* (2.77%, p < 0.05), indicating that heat treatment at different temperatures had a significant effect on enhancing the DPPH radical scavenging rate (p < 0.05). Specifically, at 70 °C treatment, the scavenging rate increased to a maximum of 74.39% on day 10; at 75 °C treatment, it peaked at 75.34% on day 8 and then decreased slightly afterward; at 80 °C treatment, it reached the highest value of 74.94% on day 2, with the DPPH radical scavenging capacity being almost completely lost from day 6 onward. Notably, the increase rate of DPPH radical scavenging capacity was higher at higher temperatures, reflecting a temperature-dependent enhancement trend in the early stage of heat treatment.

As shown in Fig. 3B, the ABTS radical scavenging ability of *A. chinense* varied with temperature and treatment duration, and at all tested temperatures, it was significantly higher than the initial scavenging rate. Specifically, at 70 °C treatment, the ABTS radical scavenging ability reached a maximum of 90.27% on day 8; at 75 °C treatment, it peaked at 90.59% on day 6; at 80 °C treatment, it reached the highest value of 88.76% on day 2. A clear temperature-dependent trend was observed: the higher the temperature, the earlier the ABTS radical scavenging ability reached its peak. Notably, compared with the 70 °C and 75 °C groups, the ABTS radical scavenging ability of the 80 °C group decreased to a certain extent after day 4, reflecting a more rapid decline in antioxidant capacity at higher temperatures following the peak.

As shown in Fig. 3C, the hydroxyl radical scavenging rate of *A. chinense* exhibited distinct patterns under different temperature treatments, with the maximum scavenging rate in each group being significantly different from that of fresh *A. chinense*. Specifically, at 70 °C treatment, the hydroxyl radical scavenging rate reached a maximum of 98.92% on day 10; at 75 °C treatment, it increased to a peak of 98.97% on day 4; at 80 °C treatment, it reached the highest value of 98.09% on day 2. Overall, the hydroxyl radical scavenging rate showed a trend of first increasing and then decreasing across all temperature groups, and a clear temperature-dependent change rate was observed: the higher the temperature, the faster the scavenging rate followed this “first rise then fall” trend.

As shown in Fig. 3D, the ferric reducing antioxidant power (FRAP) of *A. chinense* increased significantly after heat treatment at all tested temperatures compared with the fresh sample (*p* < 0.05). Specifically, at 70 °C treatment, the FRAP value reached a maximum of 42.39 μg ascorbic acid equivalents per gram (μg AAE/g) on day 10; at 75 °C treatment, it increased to a peak of 113.40 μg AAE/g on day 10; at 80 °C treatment, it reached the highest value of 105.26 μg AAE/g on day 6. A temperature-dependent change rate was evident: the higher the treatment temperature, the faster the FRAP value changed. Notably, the FRAP value of the 80 °C group began to decrease after reaching its peak, which is consistent with the trend of antioxidant-related indicators declining at higher temperatures following maximum accumulation observed in previous analyses.

Collectively, the above results demonstrate that heat treatment effectively improved the antioxidant capacity of *A. chinense*-a finding consistent with previous reports (Park & Lee, 2021). This enhancement is likely attributed to the production of phenolic compounds with antioxidant properties during heat treatment within a certain duration. Regarding the subsequent decrease in antioxidant capacity observed in the 75 °C and 80 °C groups, this phenomenon may be primarily attributed to the reduction in total phenol content (as discussed in earlier analyses), as phenolic compounds are key contributors to antioxidant activity. Additionally, it may be caused by pH changes induced by the Maillard reaction, which have been shown to modulate antioxidant capacity (Peterson et al., 2024).

### 3.9 Non-target metabolomics analysis

**Fig. 4:**
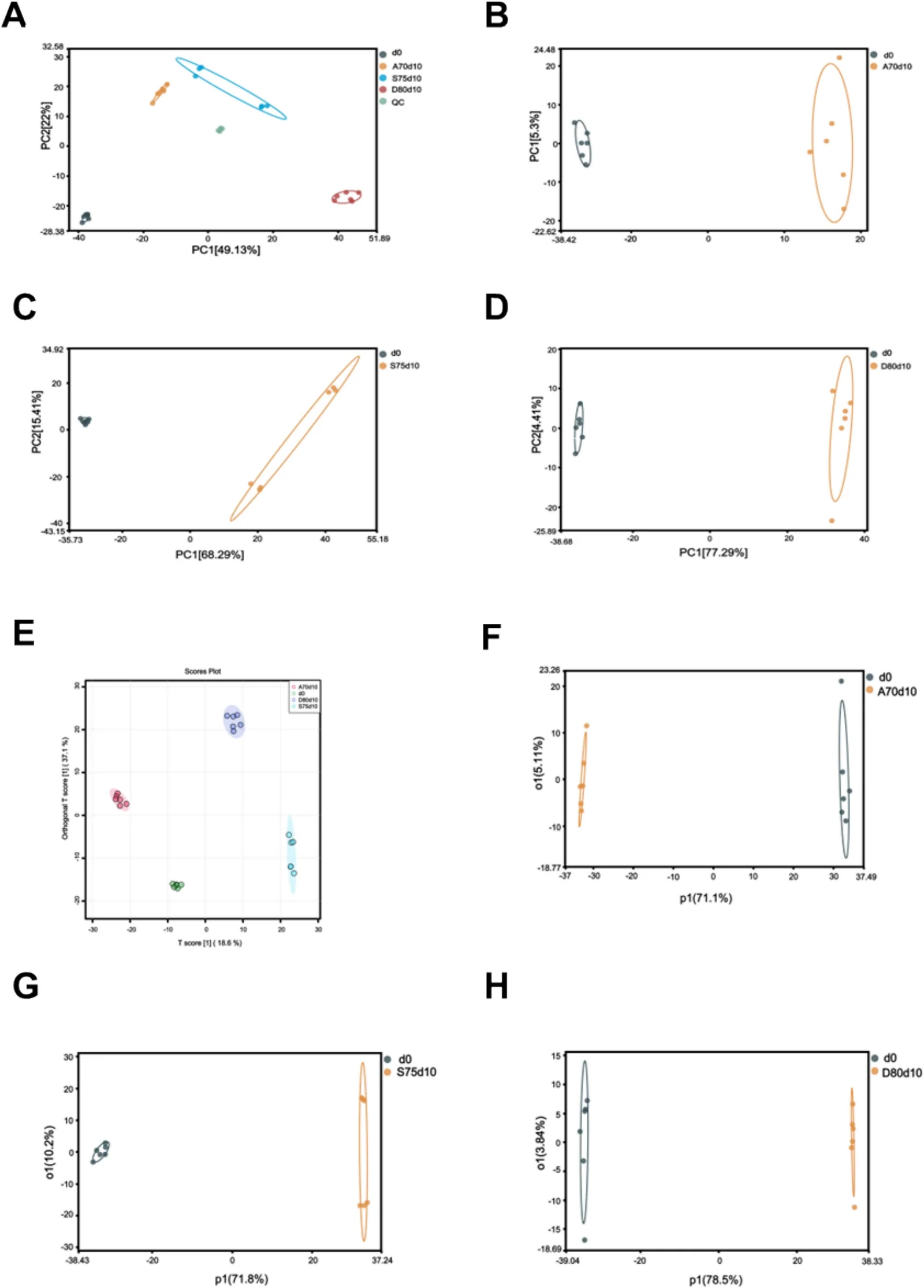
Multivariate statistical analysis of metabolomics data from fresh and blackened *A. chinense*. (A) PCA score plot: Day 0 vs 70 °C, 75 °C, and 80 °C; (B) PCA score plot: Day 0 vs 70 °C; (C) PCA score plot: Day 0 vs 75 °C; (D) PCA score plot: Day 0 vs 80 °C; (E) OPLS-DA score plot: Day 0 vs 70 °C, 75 °C, and 80 °C; (F) OPLS-DA score plot: Day 0 vs 70 °C; (G) OPLS-DA score plot: Day 0 vs 75 °C; (H) OPLS-DA score plot: Day 0 vs 80 °C. Each point represents an individual biological replicate (*n* = 6 per group).

Unsupervised principal component analysis (PCA) was conducted to compare the metabolite profiles of untreated fresh *A. chinense* and heat-treated *A. chinense* under different temperatures. As shown in Fig. 4A, B, C and D, for the four sample groups (fresh + three heat-treated groups), the contribution rate of principal component 1 (PC1) was 49.13%, principal component 2 (PC2) was 22%, and the total contribution rate of PC1 and PC2 reached 71.13%. When analyzed separately by temperature group: under 70 °C treatment, PC1 contributed 71.3%, PC2 contributed 5.3%, with a total contribution rate of 76.6%; under 75 °C treatment, PC1 contributed 68.29%, PC2 contributed 15.41%, and the total contribution rate was 83.7%; at 80 °C treatment, PC1 contributed 77.29%, PC2 contributed 4.41%, resulting in a total contribution rate of 81.70%. Notably, all total contribution rates of PC1 and PC2 exceeded 70%, indicating good explanatory power of the PCA model. Preliminary analysis results revealed significant differences in metabolite composition between the final heat-treated samples and the initial fresh sample across all temperature groups (Liang et al., 2023).

Orthogonal partial least squares-discriminant analysis (OPLS-DA) is widely used in metabolomic studies because it can more effectively exclude variables unrelated to sample classification, thereby facilitating the screening of characteristic variables for different sample groups (Huang et al., 2024; Zhang, Ross, et al., 2021). As shown in Fig. 4E, F, G and H, a significant spatial separation was observed between the blackened *A. chinense* groups (treated at different temperatures) and the fresh *A. chinense* group. This distinct separation indicates that the blackening process of *A. chinense* induced by temperature differences leads to significant changes in its metabolite composition — further confirming the inter-group differences in metabolites initially suggested by the PCA results.

#### 3.9.1 Targeted analysis of differential metabolites

**Fig. 5:**
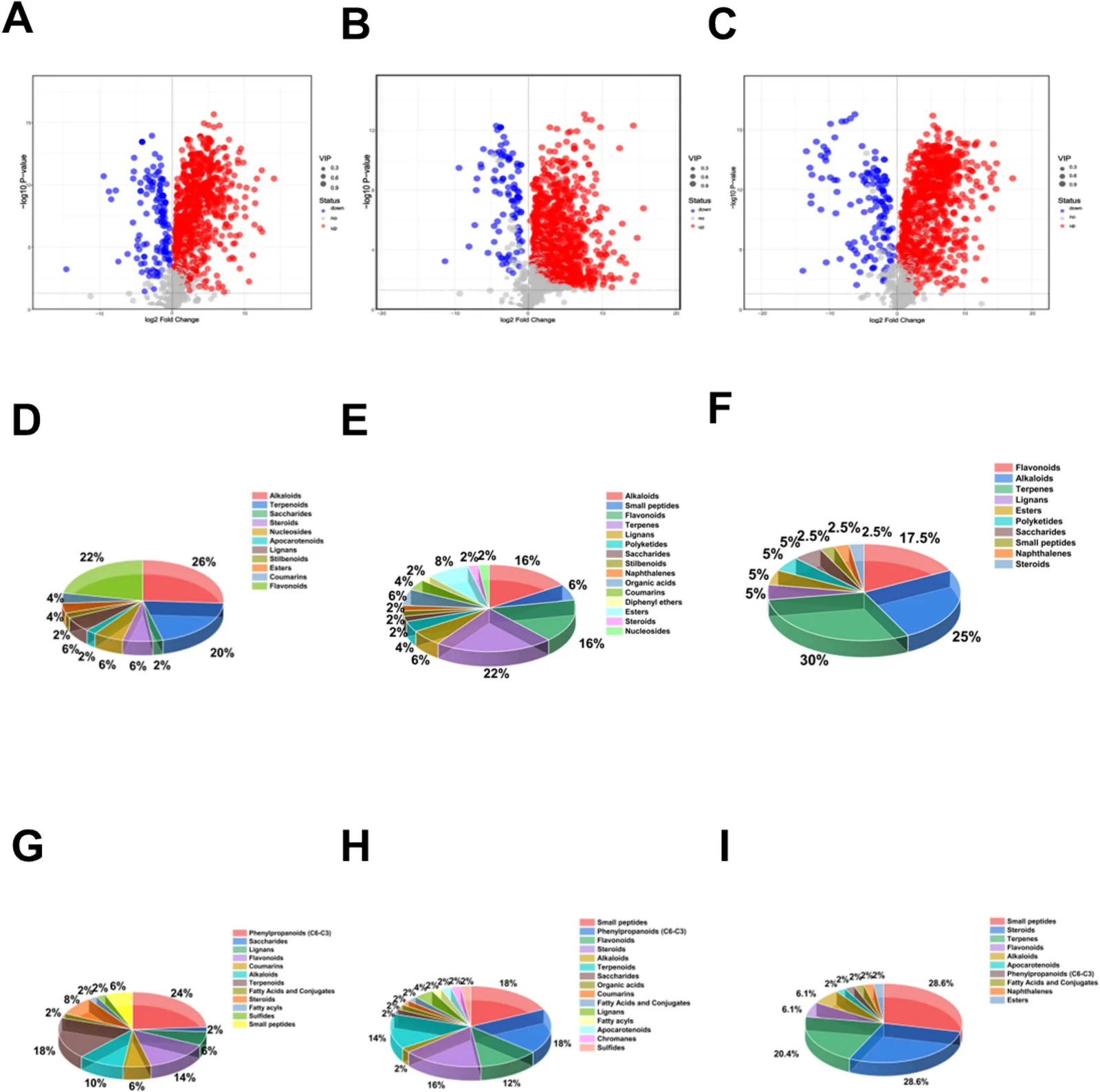
Differential metabolite analysis of fresh vs blackened *A. chinense*. (A) Volcano plot of differential metabolites: Day 0 vs 70 °C (1034 differential metabolites: 883 up-regulated, 151 down-regulated); (B) Volcano plot of differential metabolites: Day 0 vs 75 °C (1053 differential metabolites: 949 up-regulated, 104 down-regulated); (C) Volcano plot of differential metabolites: Day 0 vs 80 °C (1133 differential metabolites: 997 up-regulated, 136 down-regulated); (D) 70 °C: categories of up-regulated differential metabolites; (E) 75 °C: categories of up-regulated differential metabolites; (F) 80 °C: categories of up-regulated differential metabolites; (G) 70 °C: categories of down-regulated differential metabolites; (H) 75 °C: categories of down-regulated differential metabolites; (I) 80 °C: categories of down-regulated differential metabolites. Volcano plots show log₂(fold change) on the x-axis and –log₁₀(*p*-value) on the y-axis. Red and blue dots indicate significantly up-regulated and down-regulated metabolites, respectively (Student's *t*-test *p* ≤ 0.05, VIP ≥ 1.0, FC ≥ 2.0 or ≤ 0.5). Gray dots represent metabolites with no significant difference. Notably, γ-glutamyl-S-ally-L-cysteine (GSAC), a key pungency-related compound, exhibited a fold change of <0.2 (down-regulated) in all three temperature groups. (For interpretation of the references to color in this figure legend, the reader is referred to the web version of this article.)

Differential metabolites were screened using the following criteria: Student's *t*-test *p* ≤ 0.05 and Variable Importance in the Projection (VIP) value ≥1 (from the first principal component of the OPLS-DA model), and these differential metabolites were visualized via volcano plots. Specifically, in the 70 °C treatment group, a total of 1034 differential metabolites were identified, including 883 up-regulated and 151 down-regulated metabolites (Fig. 5A); in the 75 °C treatment group, 1053 differential metabolites were detected, with 949 up-regulated and 104 down-regulated (Fig. 5B); in the 80 °C treatment group, the number of identified differential metabolites reached 1133, consisting of 997 up-regulated and 136 down-regulated metabolites (Fig. 5C). Notably, the total number of differential metabolites increased slightly with increasing treatment temperature, with up-regulated metabolites accounting for the majority of the total (≥ 85%) in all three temperature groups.

The Human Metabolome Database (HMDB) was used to classify and analyze the top 50 up-regulated and top 50 down-regulated differential metabolites in each of the three temperature treatment groups. The results are as follows: In the 70 °C treatment group, the up-regulated metabolites primarily included alkaloids (26%), flavonoids (22%), terpenoids (20%), and other metabolites (32%) (Fig. 5D), while the down-regulated metabolites were mainly phenylpropanoids (24%) and terpenoids (18%) (Fig. 5G); in the 75 °C treatment group, the up-regulated metabolites consisted of terpenoids (22%), alkaloids (16%), flavonoids (16%), esters (8%), and other metabolites (38%) (Fig. 5E), with the down-regulated metabolites dominated by sulfur-containing amino acid small peptides (18%) and phenylpropanoids (18%) (Fig. 5H); in the 80 °C treatment group, the up-regulated metabolites were mainly terpenoids (30%), alkaloids (25%), flavonoids (17.5%), and other metabolites (27.5%) (Fig. 5F), and the down-regulated metabolites were primarily sulfur-containing amino acid small peptides (28.6%) and steroids (28.6%) (Fig. 5I). Notably, terpenoids, alkaloids, and flavonoids were the major classes of up-regulated metabolites across all three groups, with the proportion of terpenoids increasing slightly with rising temperature.

#### 3.9.2 Metabolic pathway analysis

**Fig. 6:**
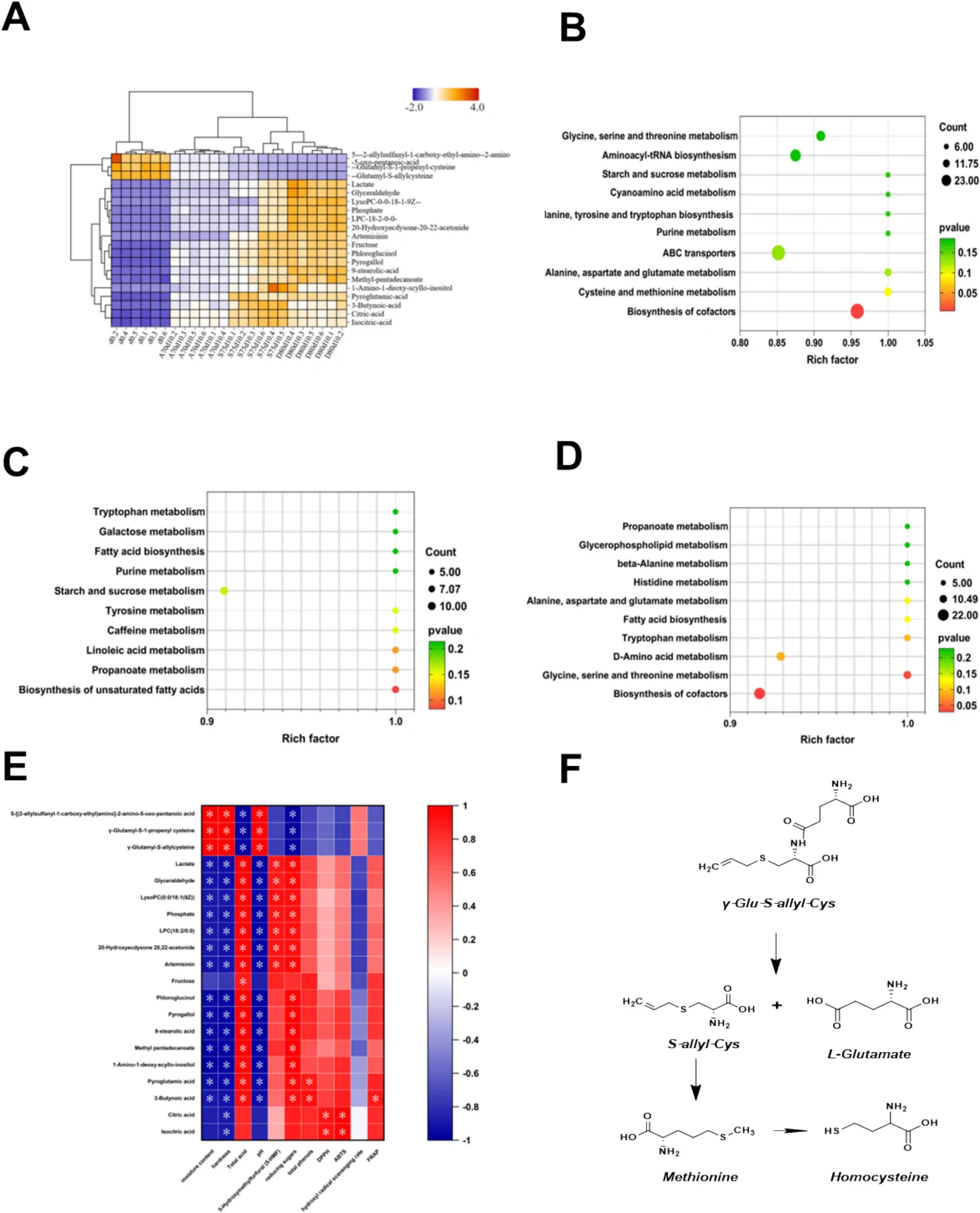
Hierarchical clustering, KEGG enrichment, and correlation analysis of differential metabolites. (A) Hierarchical clustering heatmap of the top 20 differential metabolites. Blue denotes metabolite levels below the mean, while red denotes levels above the mean.; (B) KEGG enrichment map of differential metabolites: Day 0 vs 70 °C; (C) KEGG enrichment map of differential metabolites: Day 0 vs 75 °C treatment; (D) KEGG enrichment map of differential metabolites: Day 0 vs 80 °C; (E) Correlation analysis between key quality indicators and the top 20 differential metabolites. The color scale indicates Spearman's correlation coefficient (r): red represents positive correlation, blue represents negative correlation. *p < 0.05, **p < 0.01; (F) Proposed pathway of thermal degradation of γ-glutamyl-S-allyl-L-cysteine (GSAC) during the blackening process. (For interpretation of the references to color in this figure legend, the reader is referred to the web version of this article.)

Hierarchical cluster analysis was performed on the top 20 differential metabolites in the three temperature treatment groups and the control group (fresh *A. chinense*), with the results shown in Fig. 6A. Notably, compared with the control group, three sulfur-containing compounds — γ-glutamyl-S-allylcysteine, γ-glutamyl-S-(1-propenyl)-L-cysteine, and γ-glutamyl-S-allylcysteine — were significantly down-regulated in all three treatment groups. These sulfur-containing compounds are recognized as the primary contributors to the pungent odor characteristic of *A. chinense* and other *Allium* species (Yoshimoto & Saito, 2019). Their down-regulation eliminated the unacceptable pungent odor of the treated *A. chinense*, which aligns with the research objective. Conversely, several metabolites were significantly up-regulated, including organic acids such as lactic acid, 9-stearic acid, butyric acid, pyroglutamic acid, citric acid, and isocitric acid — this up-regulation is consistent with the previously observed decrease in pH of heat-treated *A. chinense*, as organic acids are key contributors to acidity. Additionally, metabolites with potential physiological activities, such as phloroglucinol, pyrogallol, and artemisinin, were also up-regulated, further supporting the enhanced functional properties of heat-treated *A. chinense*.

KEGG enrichment analysis was carried out on the differential metabolites that had been identified, and KEGG enrichment bubble plots were generated for the top 10 metabolic pathways in each of the three temperature treatment groups. The results are as follows: In the 70 °C treatment group, 5 of the top 10 metabolic pathways were associated with amino acid synthesis or degradation, specifically the metabolic pathways identified included the metabolism of glycine, serine, and threonine; cyanoamino acid metabolic process; the biosynthesis of phenylalanine, tyrosine, and tryptophan; alanine, aspartate, and glutamate metabolism; as well as the metabolic pathway of cysteine and methionine. Three additional pathways were essential for maintaining basic biochemical processes of life activities, namely aminoacyl-tRNA biosynthesis, ABC transporters, and biosynthesis of cofactors, while the remaining two were starch and sucrose metabolism and purine metabolism (Fig. 6B). In the 75 °C treatment group, 4 of the top 10 pathways were related to lipid metabolism: biosynthesis of unsaturated fatty acids, linoleic acid metabolism, carnitine metabolism, and fatty acid biosynthesis; 4 pathways were associated with amino acid metabolism, notable metabolic pathways comprised tyrosine metabolic process; tryptophan metabolism; starch and sucrose metabolic pathway; together with the metabolism of galactose; the remaining 2 were propanoate metabolism and purine metabolism (Fig. 6C). In the 80 °C treatment group, 6 of the top 10 pathways related to amino acid metabolism included the metabolic pathway of beta-alanine; histidine metabolic process; metabolism of alanine, aspartate and glutamate; tryptophan metabolism; D-amino acid metabolic pathway; as well as the metabolism of glycine, serine and threonine; 2 pathways were associated with lipid metabolism (glycerophospholipid metabolism and fatty acid biosynthesis); and the other 2 were propanoate metabolism and biosynthesis of cofactors (Fig. 6D). The synergistic effect of these metabolic pathways enhanced the physiological activity of the treated *A. chinense;* for instance, starch and sucrose metabolism contributed to the production of sweet and reducing metabolites such as d-fructose and cellobiose (Zhang, Shan, et al., 2021). While propanoate metabolism generated propionic acid—this not only reduced the product pH but also endowed it with a certain anti-inflammatory capacity (Duan et al., 2023).

The key quality parameters at the end of the 10-day blackening process for each temperature are summarized in Table 2. In summary, based on the comprehensive evaluation of physicochemical properties, antioxidant activities, and metabolite profiles, blackening at 75 °C is proposed as the optimal condition. This temperature yielded the highest total phenolic content (94.54 mg GAE/g), sustained high antioxidant capacity across multiple assays, substantial accumulation of desirable metabolites, and effective reduction of pungent sulfur compounds, while maintaining favorable texture and color development.

**Table 2:** Summary of key quality parameters of blackened *A. chinense* after 10 days of treatment at different temperatures.

Parameter	70 °C (Day 10)	75°C (Day 10)	80 °C (Day 10)
TPC (mg GAE/g)	30.78	94.54	35.67
5-HMF (μg/g)	23.86	166.37	318.14
DMs (up/down)	883/151	949/104	997/136
DPPH (%)	74.39	66.73	1.43
FRAP (μg AAE/g)	42.39	113.40	37.11

**Note:** TPC, total phenolic content; 5-HMF, 5-hydroxymethylfurfural; DMs, differential metabolites; DPPH, 2,2-diphenyl-1-picrylhydrazyl radical scavenging activity; FRAP, ferric reducing antioxidant power. Bold values indicate the optimal performance at 75°C. The 80 °C TPC value at Day 10 (∼70 mg GAE/g) represents a decline from its peak (94.38 mg GAE/g at Day 4) due to thermal degradation.

While our metabolomics data reveal significant temperature-dependent changes in metabolites with known bioactivities, it is important to note that the observed correlations between metabolite changes and biological effects are associative rather than causal. The up-regulation of terpenoids, flavonoids, and organic acids aligns with enhanced in vitro antioxidant activity, but direct evidence of their in vivo bioavailability and physiological effects is required to confirm their functional significance (Reena et al., 2022). Similarly, the reduction in γ-glutamyl peptides is strongly associated with decreased pungency potential based on established biochemical pathways (Yoshimoto & Saito, 2019), but formal sensory validation using trained panels would be necessary to definitively establish this relationship. Future studies employing targeted bioassays, in vivo models, and sensory evaluation are warranted to validate the hypothesized biological activities of the identified metabolites.

### 3.10 Correlation analysis

It is important to note that correlation analysis demonstrates association between variables but does not establish causation. The observed correlations provide hypotheses for mechanistic relationships that require further experimental validation. Correlation analysis was performed between the 20 metabolites with the largest fold change (FC) values in the final products of the three treatment groups and the indicators measured in the previous study. As shown in Fig. 6E, 1-amino-1-deoxy-scyllo-inositol exhibited a strong negative correlation with water content and hardness. As an inositol derivative, this compound is commonly used as an osmotic pressure protector, which can maintain the osmotic pressure balance in cells and prevent water loss (Chhetri, 2019). A strong negative correlation was observed between moisture content and most metabolites (e.g., 5-[(2-allylsulfanyl-1-carboxy-ethyl) amino]-2-amino-5-oxo-pentanoic acid and γ-Glutamyl-S-1-propenyl cysteine), indicating that a reduction in moisture content elevated the levels of these metabolites in the present study. γ-Glutamyl cysteine derivatives act as flavor precursors and antioxidant cofactors, and also exhibit anti-inflammatory properties. This provides a metabolic basis for the transition from “low moisture content to high functional activity” in the samples (Y. Yang et al., 2019). Additionally, three metabolites — 5-[(2-allylsulfanyl-1-carboxy-ethyl) amino]-2-amino-5-oxo-pentanoic acid, γ-glutamyl-S-1-propenyl cysteine, and γ-glutamyl-S-allylcysteine-showed strong negative correlations with total acid and reducing sugar contents, as well as strong positive correlations with pH (*p* < 0.01).

More importantly, the observed correlations point to underlying mechanistic pathways linking metabolite shifts to enhanced antioxidant activity. Among the top correlations identified, citric acid and DPPH radical scavenging rate exhibited a strong positive correlation (*r* = 0.91, *p* < 0.05), and isocitric acid showed a similarly strong positive correlation with ABTS radical scavenging rate (*r* = 0.97, p < 0.05). These correlations suggest a potential mechanistic link through the dual role of these organic acids in the TCA cycle and metal chelation. As key intermediates of the tricarboxylic acid (TCA) cycle, citric acid and isocitric acid sustain cellular energy metabolism, which indirectly supports the regeneration of antioxidant systems (e.g., the glutathione cycle) by providing reducing equivalents in the form of NADH. Their carboxyl groups can also chelate pro-oxidant metal ions such as Fe^2+^ and Cu^+^, thereby inhibiting Fenton reaction-mediated hydroxyl radical generation (Reena et al., 2022). This metal-chelating property is particularly relevant given that TCA cycle intermediates are generated in abundance during the blackening process, as confirmed by the up-regulation of organic acids in our metabolomics data (Fig. 6A). The correlation between TCA cycle metabolites and antioxidant activity provides a mechanistic hypothesis linking primary metabolism and functional quality enhancement in blackened *A. chinense.*

Similarly, the positive correlations between total phenol content/antioxidant indices and metabolites such as fructose and pyrogallol suggest synergistic pathways. Fructose, as a reducing sugar, participates in the Maillard reaction to generate melanoidins and other advanced glycation end-products that possess radical-scavenging activity. Pyrogallol, a polyphenolic compound, directly donates electrons or hydrogen atoms to neutralize free radicals, and its presence may also promote the polymerization of phenolics into higher molecular weight antioxidants (Hamed et al., 2024; Lee et al., 2016). Conversely, the contents of metabolites such as γ-Glu-S-allyl-Cys exhibited negative correlations with antioxidant indices. These compounds were decomposed under thermal treatment to generate downstream metabolites that participate in the methionine and cysteine metabolic pathways, shifting from pungency-related pathways toward the accumulation of antioxidant-friendly metabolites (Fig. 6F). Such intercorrelated metabolic remodeling—characterized by reduced pungent sulfur compounds and enhanced accumulation of organic acids and phenolic derivatives — collectively suggests a biochemical basis for the improved antioxidant capacity of processed black *A. chinense*.

The blackening process of *A. chinense* involves complex physicochemical transformations that are fundamentally distinct from those in other Allium species, owing to its unique sulfur-containing metabolite profile dominated by γ-glutamyl peptides rather than alliin. In black garlic (*Allium sativum*), the Maillard reaction drives color development, flavor formation, and bioactive compound generation, converting pungent organosulfur compounds such as allicin into stable S-allyl-L-cysteine and other derivatives that contribute to the mild flavor of the final product. Similar temperature-dependent transformations—including increased total acid, reducing sugars, polyphenols, and 5-HMF alongside enhanced antioxidant activity—have been documented in black jujube (Song et al., 2022). However, the metabolic pathways governing the blackening process in *A. chinense* remain largely unexplored. Our findings provide the first comprehensive metabolomic characterization of temperature-dependent metabolic reprogramming during *A. chinense* blackening, revealing both conserved and unique transformation patterns compared to other blackened products.

## 4 Conclusion

This study demonstrates that heat-induced blackening effectively enhances the flavor, odor, and nutritional quality of *A. chinense*. The process improved product color and texture, and significantly increased total acidity, reducing sugar content, total phenolic content, and antioxidant capacity. Metabolomics analysis further revealed a marked reduction in sulfur-containing compounds, which contributed to the decrease in pungency, alongside the accumulation of functionally active metabolites such as terpenoids, alkaloids, and flavonoids. Notably, based on the comprehensive evaluation of physicochemical properties, antioxidant activities, and metabolite profiles, blackening at 75 °C is proposed as the optimal condition. This temperature yielded the highest total phenolic content (94.54 mg GAE/g), sustained high antioxidant capacity across multiple assays, substantial accumulation of desirable metabolites, and effective reduction of pungent sulfur compounds, while maintaining favorable texture and color development. Furthermore, this study provides a scientific foundation for developing novel blackened *A. chinense* products. However, several limitations of the present study should be acknowledged. First, while our in vitro assays demonstrate significant antioxidant activity, the absence of in vivo validation (e.g., animal models or human trials) limits the translational relevance of these findings, as the bioavailability and actual physiological effects of the identified bioactive compounds remain unknown. Second, although the reduction of sulfur-containing compounds was correlated with decreased pungency, formal sensory evaluation using trained panels is needed to definitively validate the flavor improvement claims. Third, while the laboratory-scale process (10 days at 75 °C) yielded optimal product quality, industrial-scale feasibility—including energy costs, batch-to-batch consistency, and economic viability—has not been assessed. Nevertheless, the 10-day process is shorter than traditional black garlic (60–90 days) and comparable to optimized processes (8–15 days), suggesting favorable industrial potential. Notably, this is the first report on *A. chinense* blackening. Future research should focus on the long-term storage stability, in vivo bioavailability assays and pharmacokinetic studies, and scaling-up of the blackening process to facilitate its industrial application and functional food development.

## CRediT authorship contribution statement

**Junxiong Hao:** Writing – original draft, Validation, Methodology, Formal analysis, Data curation, Conceptualization. **Yingying Yang:** Validation, Investigation. **Jie Liu:** Investigation, Data curation. **Fei Shen:** Investigation, Data curation. **Ying Chen:** Investigation, Data curation. **Xiaoxia Zuo:** Validation, Supervision. **Jing Wang:** Supervision, Investigation. **Liqin Zhu:** Supervision, Project administration. **Yonggen Shen:** Validation, Supervision. **Rui Shi:** Validation, Data curation. **Hongliang Yao:** Investigation, Data curation. **Libin Wang:** Validation, Data curation. **Zhipeng Cai:** Writing – review & editing, Supervision, Resources, Funding acquisition, Conceptualization.

## Funding

This work was supported by the 10.13039/501100001809National Natural Science Foundation of China (31902128), and the Teaching Reform Research Project of Jiangxi Agricultural University (2022B2SZ04).

## Declaration of competing interest

The authors declare that they have no known competing financial interests or personal relationships that could have appeared to influence the work reported in this paper.

## Data Availability

Data will be made available on request.
