# Supplementary material for: Heat-induced blackening enhances antioxidants and reduces pungency in Allium chinense: Metabolomics insights
Source: Food Chem X. 2026 Aug 12;38:104293. doi: 10.1016/j.fochx.2026.104293 (PMC13503101; doi:10.1016/j.fochx.2026.104293)
Supplement: Supplementary file 1 [file mmc1.docx]

**Heat-Induced Blackening Enhances Antioxidants and Reduces Pungency in *Allium chinense*: Metabolomics Insights**

Junxiong Hao^a^, Yingying Yang^a^, Jie Liu^a^, Fei Shen^a^, Ying Chen^a^, Xiaoxia Zuo^a^, Jing Wang^a^, Liqin Zhu^a^, Yonggen Shen^a^, Rui Shi^b^, Hongliang Yao^c^, Libin Wang^b,d^, Zhipeng Cai^a,e,*^

a: College of Food Science and Engineering, Jiangxi Agricultural University, Nanchang, Jiangxi 330045, China

b: Department of Food Science and Technology, College of Light Industry and Food Engineering, Nanjing Forestry University, Nanjing, Jiangsu 210037, China

c: College of Animal Science and Food Engineering, Jinling Institute of Technology, Nanjing, Jiangsu 210038, China

d: National Key Laboratory of Crop Genetics and Germplasm Enhancement and Utilization, Sanya Institute of Nanjing Agricultural University, College of Horticulture, Nanjing Agricultural University, Nanjing, Jiangsu 210095, China

e: Jiangxi Wanzai Food Processing Science and Technology Backyard, Yichun, Jiangxi 336100, China

* Corresponding author. College of Food Science and Engineering, Jiangxi Agricultural University, Nanchang, Jiangxi 330045, China

E-mail addresses: caizhipeng-sp@jxau.edu.cn (Zhipeng Cai)

Supplementary Fig. S1. Representative HPLC chromatograms of 5-HMF standards and blackened *A. chinense* samples. (A-F) 5-HMF standard solutions at concentrations of 0.5, 2.5, 5.0, 10.0, 50.0, and 100.0 μg/mL; (G) Representative chromatogram of a blackened *A. chinense* sample (75 °C, Day 10). The 5-HMF peak was identified at a retention time of approximately 11.5 min (indicated by arrow). LC conditions: Inertsustain^®^ C18 column (4.6 mm × 250 mm, 5 μm), column temperature 35 °C, mobile phase 0.1% formic acid in water : acetonitrile (95:5, v/v), flow rate 1.0 mL/min, injection volume 10 μL, detection wavelength 284 nm, total runtime 20 min.
